# Preparation of Fluorescent Carbon Dots Composites and Their Potential Applications in Biomedicine and Drug Delivery—A Review

**DOI:** 10.3390/pharmaceutics14112482

**Published:** 2022-11-16

**Authors:** Yaru Chai, Yashan Feng, Kun Zhang, Jingan Li

**Affiliations:** 1 School of Materials Science and Engineering, Zhengzhou University, Zhengzhou 450000, China; 2 Advanced Functional Materials Laboratory, Zhengzhou Railway Vocational & Technical College, Zhengzhou 450000, China; 3 School of Life Science, Zhengzhou University, Zhengzhou 450000, China

**Keywords:** carbon dots, composite materials, synthesis, bioapplication, capacitor, electrocatalysis, photocatalysis

## Abstract

Carbon dots (CDs), a new member of carbon nanostructures, rely on surface modification and functionalization for their good fluorescence phosphorescence and excellent physical and chemical properties, including small size (<10 nm), high chemical stability, biocompatibility, non-toxicity, low cost, and easy synthesis. In the field of medical research on cancer (IARC), CDs, a new material with unique optical properties as a photosensitizer, are being applied to heating local apoptosis induction of cancer cells. In addition, imaging tools can also be combined with a drug to form the nanometer complex compound, the imaging guidance for multi-function dosage, so as to improve the efficiency of drug delivery, which also plays a big role in genetic diagnosis. This paper mainly includes three parts: The first part briefly introduces the synthesis and preparation of carbon dots, and summarizes the advantages and disadvantages of different preparation methods; The second part introduces the preparation methods of carbon dot composites. Finally, the application status of carbon dot composites in biomedicine, cancer theranostics, drug delivery, electrochemistry, and photocatalysis is summarized.

## 1. Introduction

**Figure 1:**
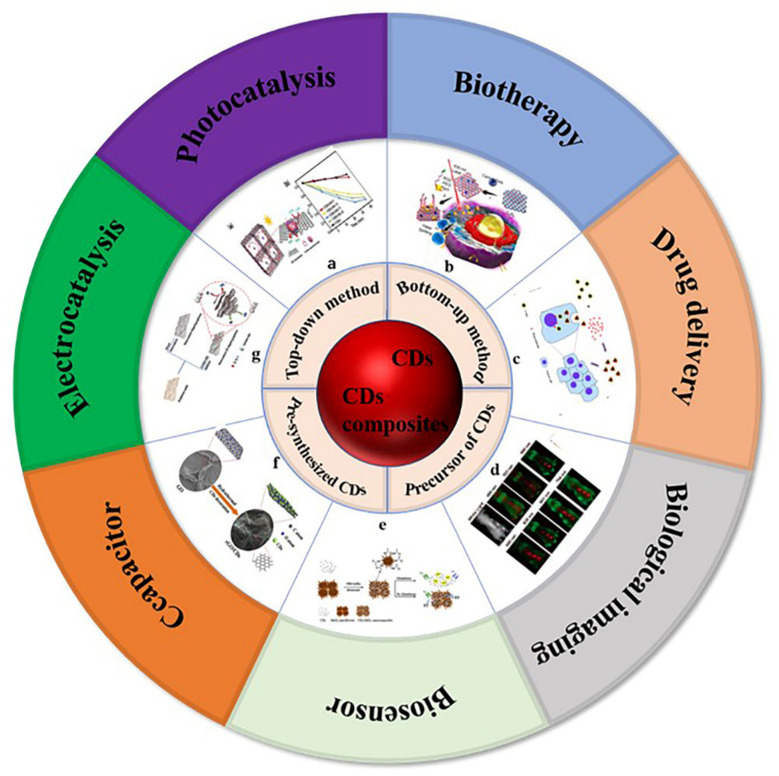
Preparation methods and application classification of carbon dots and carbon dots composites. (**a**) Photocatalysis: Photocatalytic mechanism of 3D CQDs/GA composites, photocatalytic reduction of Cr (VI) with different proportions of CQDs/GA and original CQD [4]. (**b**) Biotherapy: Structure of PCCN and schematic diagram of 630 nm light-driven water splitting enhanced PDT [5]. (**c**) Drug delivery: Image of fluorescent carbon nanoparticles in medical cancer treatment [6]. (**d**) Biological imaging: In vivo imaging and biodistribution of the carboxylated Graphene Quantum Dots [7]. (**e**) Biosensor: Schematic diagram of GSH detection of CD-MnO_2_ nanocomposites [8]. (**f**) Capacitor: Schematic diagram of synthesis process of three-dimensional interconnected CD decorative reduced graphene oxide nanosheets (rGO/CDs) [9]. (**g**) Electrocatalysis: Schematic preparation process of NS-CD@gf [10].

Carbon is the most basic element in organic matter and exists in the form of different compounds. Carbon dots (CDs) are a new carbon based zero-dimensional nanomaterial [1]. During the process of separation and purification of single-walled carbon nanotubes (SWCNTs) by gel electrophoresis, Sun et al. used laser ablation to make a series of surface treatments on the obtained carbon nanoparticles and named them carbon dots [2]. CDs are a large number of different, quasi spherical carbon atom aggregation regions with a size of less than 10 nm. CDs are usually composed of sp2 and sp3 hybrid carbon nuclei and rich active functional groups (such as hydroxyl, carboxyl, amino, etc.), which makes them have excellent water solubility. Through a series of chemical reactions, small molecules, organic polymers, or biomolecules can be adsorbed on the surface of CDs for surface passivation or functionalization, but the quantum yield of CDs is low, which greatly limits their practical application in the development of biological imaging and therapeutic diagnostics. In addition, because there is no specific analyte recognition group on the surface of CDs, the detection process of CDs in biosensors is often affected by potential disruptors. Most importantly, the interaction between CDs and biological systems is usually poor and lacks specificity, which greatly limits their potential clinical application. Generally, surface passivation can enhance the luminescence properties of CDs, and surface functionalization can change the physicochemical properties of CDs [3]. The preparation method and application classification of carbon dots and carbon dot composites are shown in Figure 1 [4,5,6,7,8,9,10]. Carbon dot matrix composites have excellent luminescence properties and good biocompatibility, and they have attracted extensive attention in the fields of biological imaging [11,12,13], sensing [14,15,16], detection, and biochemical analysis.

## 2. Synthesis of Carbon Dots

**Figure 2:**
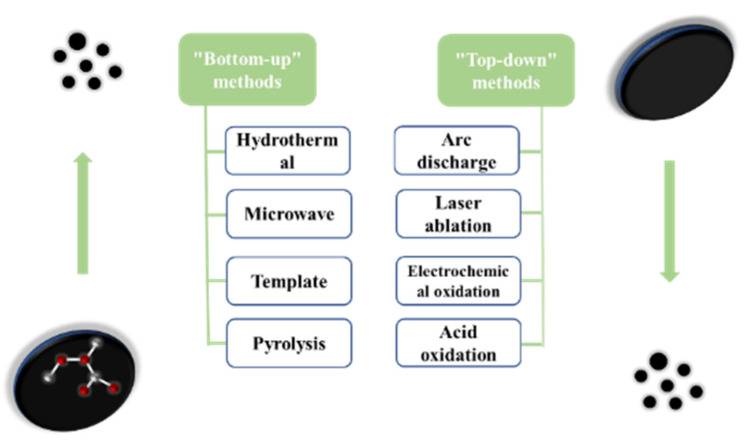
The two preparation methods of carbon dots.

There are many studies on the different synthesis methods of carbon dots. Each process aims to improve the synthesis strategy and optimize the reaction conditions so that the carbon dots are not only more cost-effective and eco-friendly, but also provide more excellent performance. According to the carbon source and the used process, the synthesis methods are mainly divided into the “top-down” method and “bottom-up” method (as shown in Figure 2).

### 2.1. “Top-Down” Method

The “top-down” method is a method of peeling or breaking large carbon materials to form small carbon nanoparticles, and then modifying their surfaces to improve their luminous efficiency, mainly including arc discharge method [17], laser ablation [18], electrochemical oxidation [19], and acid oxidation.

#### 2.1.1. Arc Discharge Method

The earliest method to prepare carbon dots is the arc discharge method, which means that under a certain voltage condition, gaseous charged particles are used as conductors, and the reaction is accelerated by generating a strong current and high temperature. The advantage is that the prepared carbon dots have a small particle size and high oxygen content; the disadvantage is that the components of arc discharge are complex and there are many impurities, so the prepared carbon dots are difficult to separate and purify, the yield is very low, and the fluorescence quantum efficiency is also very low.

Research evolution: Xu et al. prepared single wall carbon nanotubes by the arc discharge method and separated the mixed products by electrophoresis. In this process, carbon dots were found [17]. In 2006, Bottini et al. [20] removed the fluorescent nanoparticles from the original carbon nanotubes and the fluorescence emission wavelength of the fluorescence nanoparticles gradually redshifted with the increase of the molecular weight of the nanoparticles, and the fluorescence emission from blue to yellow-green carbon points were obtained.

#### 2.1.2. Laser Ablation

Laser ablation refers to the rapid laser passivation of carbon nanoparticles in organic solvents. The advantage is that the prepared carbon dots have bright, tunable, and stable photoluminescence properties; the disadvantage is that the carbon dots prepared by laser ablation usually need irradiation, oxidation, and passivation processes. The preparation method is complex, the output of carbon dots is low, the particle size distribution is uneven, and the purity is low.

Research evolution: Sun et al. prepared fluorescent carbon dots with good luminescence properties by laser ablation with graphite powder as the carbon source [21]. Firstly, graphite powder and cement were dried and solidified to make the graphite target, which was then annealed in a high-purity Ar atmosphere. Then, the graphite target was bombarded with Nd:YAG solid-state laser at 900 °C and 75 kPa, and then refluxed with concentrated nitric acid for 12 h. Finally, the surface of the previously prepared carbon nanoparticles was passivated using some simple organic substances to obtain fluorescent carbon dots. The fluorescence properties of the carbon dots prepared by this method depend on the surface post modified groups, and the emission wavelength is excitation dependent. By adjusting the excitation wavelength, the full band emission from red to blue light can be realized. Hu et al. reported an effective method to synthesize carbon dots by laser irradiation of carbon materials suspended in organic solution [22]. Firstly, a certain amount of graphite powder is dispersed in polyethylene glycol to form a black suspension, and then a Nd:YAG pulse laser with a wavelength of 1.064 mm is used. Finally, the suspension after laser irradiation was further separated and purified to obtain carbon dots.

#### 2.1.3. Electrochemical Oxidation

Electrochemical oxidation is a method of preparing carbon quantum dots by electrolyzing some carbon materials, such as carbon nanotubes and graphite. The advantage is that the size and luminescence properties of the carbon dots can be adjusted by changing the current intensity, the preparation cost is low, and the yield is high; The disadvantage is that the fluorescence quantum efficiency of the product is low.

Research evolution: Zhou et al. prepared carbon dots by electrochemical synthesis for the first time. Firstly, the composite of multi walled carbon nanotubes and carbon film was prepared as the working electrode, Ag/AgClO_4_ as the reference electrode, and Pt wire as the counter electrode to form a three-electrode system [23]. An appropriate amount of tetrabutylammonium perchlorate was dissolved in an acetonitrile solution and used as an electrolyte after complete dissolution. The appropriate test conditions for a cyclic voltammetry test of the whole system are selected. In this process, the acetonitrile solution of tetrabutylammonium perchlorate changes from colorless to yellowish brown. After irradiation by UV lamp, it can emit blue fluorescence. Finally, it is separated and purified to obtain carbon dots with uniform size. Bao et al. proposed a new strategy to controllably prepare luminescent carbon dots by etching carbon fibers using the electrochemical method [24]. In this study, the target carbon dots could be prepared controllably only by adjusting the applied voltage.

#### 2.1.4. Acid Oxidation

The acid oxidation method consists mainly of acid treatment of the carbon source to oxidize the functional groups on the surface of carbon source to generate fluorescence. The advantages are the simple preparation method and low requirements for the experimental equipment; The disadvantage is that the yield of preparing carbon dots is generally low, the separation is difficult, and the carbon dots generally require post-treatment passivation, poor control of particle size, a violent reaction process, harsh conditions, and many steps.

In 2018, the N-CQDs, S-CQD, and Se-CQDs obtained by Iannazzo et al. showed tunable photoluminescence performance, higher quantum yield (QY), and longer fluorescence lifetime than pure CQDs [25]. The experimental results show that heavily doped heteroatoms will affect the photoluminescence characteristics, which is positively correlated with the electronegativity of N, S, and Se. The active heteroatoms on the surface of the CQDs will adjust the electronic structure of the corresponding CQDs. Therefore, when used as an electrocatalyst, it will have good electrocatalytic activity. On the other hand, the heavily doped CQDs have the ability to coordinate with transition metal ions. N-CQDs, S-CQD, and Se-CQDs may also have the potential to absorb other metal ions, such as Fe^3+^, CO^2+^, and Ni^2+^, to form so-called monatomic catalysts.

### 2.2. “Bottom-Up” Method

The “bottom-up” method is used mainly to carbonize and assemble the molecular precursors (such as sugars, organic compounds, ethylenediamine, etc.) to synthesize carbon dots through combustion or heat treatment, and the methods used are mainly the hydrothermal method [26], microwave method [27], template method, etc.

#### 2.2.1. Water/Solvothermal Method

The hydrothermal method is used to prepare carbon dots by mixing carbon source and solvent and heating in autoclave. It is worth mentioning that the hydrothermal method is the most commonly used method to prepare carbon dots at present. Reaction temperature and reaction time are the two most important parameters, which directly affect the optical properties and quantum yield of CDs. The advantages are that the source of raw materials is very wide, the reaction equipment is simple, and the reaction conditions are easy to control. Because the reaction is carried out in a closed reactor, the influence of impurities in the air is avoided, the prepared carbon dots are relatively uniform, and most of the carbon dots synthesized by the hydrothermal method have good water solubility. The disadvantage is that due to the limited volume of the hydrothermal kettle, the amount of carbon dots prepared at one time is limited, and the prepared carbon dots will produce more impurities, which makes it difficult to separate and purify the carbon dots; it is therefore not suitable for large-scale industrial production, and the size of the prepared CDs is difficult to control, which also limits the use of this method to a certain extent. In addition, due to the use of organic solvents, the solvothermal method is more dangerous than the hydrothermal method at high temperature, and the post-treatment of excessive organic solvents after reaction also needs special consideration.

Research Evolution: Peng et al. first reported the hydrothermal synthesis of fluorescent carbon dots in 2009 [28]. In this study, they first dehydrated carbohydrates with concentrated sulfuric acid to produce carbonaceous substances, then treated carbonaceous substances with nitric acid to oxidize them, and finally modified them with nitrogen-containing groups into carbon dots emitting blue fluorescence. However, the quantum yield of carbon dots synthesized by this method is not high, and the size distribution is uneven. Subsequently, the researchers found that in the process of hydrothermal synthesis of carbon dots, the size, surface chemical state, and luminescence wavelength of carbon dots can be achieved by adjusting the reaction conditions such as the type of carbon source and solvent. In 2013, Zhu et al. synthesized high fluorescence carbon dots using the hydrothermal method, and the proposed citric acid and ethylenediamine system is very classic in the field of carbon dots [29]. Citric acid and ethylenediamine undergo condensation reaction under the action of aqueous solvent to form polymer-like carbon dots, which are further carbonized to form carbon dots with an average size of 2–6 nm. The carbon dots prepared by this method have uniform size distribution, and their luminous effect is comparable to that of fluorescent dyes. In 2015, Jiang et al. used o-diphenylamine, p-Diphenylamine, and m-diphenylamine as carbon sources to synthesize carbon dots emitting red, green, and blue fluorescence in the ethanol solvent by the solvothermal method [30]. In this study, the isomers of phenylenediamine were used as carbon sources to react with the ethanol solvent. The mixed products were separated by silica gel column to obtain carbon dots with different emission wavelengths. In 2017, Raji et al. synthesized carbon dots using monk fruit as the carbon source using the hydrothermal method. The monk fruit was sealed in an autoclave, heated at 180 °C for 6 h, and the resulting solution was cooled to room temperature. The solution changed from light yellow to dark brown. The monk fruit was dehydrated, polymerized, and carbonized to finally form carbon dots with blue fluorescence [26].

#### 2.2.2. Microwave Method

Microwave heating is a method that uses a microwave to raise the temperature rapidly in a short time to polymerize and carbonize the reactant monomers to form carbon dots. It provides a new and rapid method for the synthesis of carbon dots. Experiments show that constant high-pressure reaction vessel is the best method to control the particle size distribution and photoluminescence properties of CDs [31]. The advantages are that it is convenient and fast, the preparation conditions are simple, raw materials are easily available, etc. The disadvantage is that the reaction process is unstable, the reaction temperature is difficult to control, and the fluorescence quantum yield is low.

Research evolution: In 2009, Zhu et al. synthesized carbon dots under microwave-assisted reaction conditions with polyethylene glycol and sugars as the carbon sources [32]. They put all the reactants in the microwave oven and set the power to 500 W and the reaction time to 2–10 min. It could be observed that the color of the reaction solution experienced a change of colorless to a yellow dark-brown. Finally, the blue fluorescent carbon dots were obtained through separation and purification. By changing the reaction time, the size, emission peak position and quantum yield of carbon dots can effectively be controlled. In 2012, Tang et al. synthesized carbon dots emitting deep UV fluorescence using glucose as the carbon source by microwave-assisted hydrothermal synthesis [33]. In 2015, Pan et al. used formamide and citric acid as the carbon sources and microwave-heated them at 160 °C for 60 min. After separation and purification, they obtained fluorescent carbon dots with full-spectrum emission [30]. In 2020, Li et al. used guanine and ethylenediamine as the carbon sources and deionized water as the solvent, heated it in a household microwave oven with a set power of 700 W for 10 min, and removed large impurities with a dialysis bag to obtain an aqueous solution of carbon dots [26]. The carbon dots prepared by this method have excellent water solubility, excellent stability at different pH, and extremely high ionic strength, and have been successfully used as fluorescent probes to detect Ag^+^.

#### 2.2.3. Template Method

The advantage of template synthesis of carbon dots is that the size of the carbon dots can be controlled by the size of template, and the aggregation of the carbon dots can be reduced in the process of synthesis. The prepared carbon dots have a uniform size and high fluorescence quantum yield; The disadvantage is that some templates are difficult to separate from the carbon dots, and the fluorescence performance of the carbon dots may be affected in the process of acid-base etching or heating to remove templates. The advantages are that the size of the carbon dots can be controlled by the size of the template, and the aggregation of the carbon dots can be reduced in the process of synthesis. The prepared carbon dots have a uniform size and high fluorescence quantum yield. The disadvantage is that some templates are difficult to separate from the carbon dots, and the fluorescence performance of the carbon dots may be affected in the process of acid-base etching or heating to remove the templates.

Research evolution: In 2012, Kwon et al. first synthesized fluorescent carbon dots with a size of 1.403 ± 0.148 nm using glucose as the carbon source by using the micro lotion template method [34]. Firstly, the water in the oil microemulsion was formed by mixing 10% glucose aqueous solution with 1-octanol in oil phase. Then, cetylammonium was added for heating and carbonization. Finally, cetylammonium modified fluorescent carbon dots were obtained. In this process, the number of carbon sources in each micelle can be limited by adjusting the concentration of micro lotion, and the size of carbon dots can be further controlled. In 2013, Yang et al. first proposed the method of soft hard template, using copolymer P123 as the soft template and ordered mesoporous silica as the hard template to prepare fluorescent carbon dots [35].

## 3. Synthesis of CDs Composites

**Figure 3:**
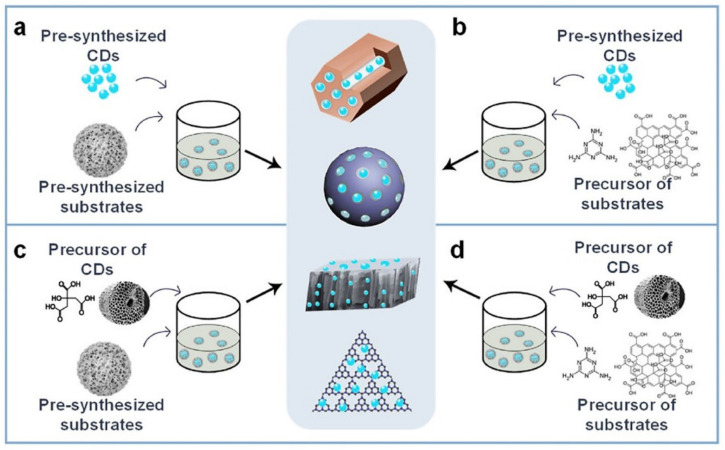
Illustration of CDs composite materials synthesized using different approaches [41]: (**a**) Prepare CDs and substrate materials respectively, and then combine them to form CDs composites; (**b**) Prepared CDs as one of the raw materials to prepare the substrate material; (**c**) Add the prepared substrate material to the preparation process of CDs to form CDs; (**d**) One-step method to prepare CDs composites.

Carbon dots have good water solubility, fluorescence, biocompatibility, and low cost. However, their small volume and aggregation luminescence quenching hinder their further application in some fields. Therefore, carbon dot composites are further promoted and utilized. Carbon dots can be combined with some base materials to form nanowires [36], nanotubes [37], particles [38], films, sols [39], and three-dimensional gel [40]. In general, four methods are usually used to prepare CDs composites, as shown in Figure 3 [41].

The first method is to prepare the CDs and substrate materials, respectively, and then combine them to form CDs composites. It can protect the stability and initial properties of CDs and substrate materials, so it has been widely used. In the past few years, many research groups have modified CDs onto substrate materials for further application. In order to retain the initial properties of CDs and substrate materials, researchers mixed them together, and then loaded CDs onto substrate materials by electrodeposition [42], ultrasonic crushing [43,44,45], impregnation [46,47,48], and heat treatment [49,50,51] (Figure 3a). The synthesized composites not only have the initial properties of CDs and substrate, but also have the reinforcement properties in other application fields. The interaction between CDs and substrate is usually electrostatic force [52,53], capillary force, charge transfer [54], and stacking force [55]. Benetti et al. prepared a CDs-based graphene oxide hybrid on perovskite solar cell (PSC). The charge transfer interaction between cadmium and graphene oxide leads to obvious photo-quenching. After the optimization of CDs content, the PSC efficiency was increased from 14.7% to 16.2%.

Zhang et al. invented a CdS/MoSx composite, which provides a new way for the photocatalytic hydrogen production technology of CDs [56]. The controllable synthesis of CdS/MoSx can be realized by electrochemical deposition with self-supporting materials as the substrate, using an electrolyte containing a cadmium source, molybdenum source, and sulfur source, using transition metal sulfide MoSx instead of noble metal as the cocatalyst, and fixing CdS/MoSx on the substrate by electrochemical one-step electrodeposition. The prepared CdS/MoSx composites can make full use of the visible light, increase the conversion utilization of solar energy, have a good light response to the visible light region, and have better photocatalytic activity.

The second method is to use the prepared CDs as one of the raw materials to prepare the substrate material, so as to form CDs composites. This is also a general method, and the preparation process is simple. Method 2 in Figure 3b is another common method for preparing CDs composites. Firstly, CDs are prepared using the above method, and then the CDs are used as the raw material for substrate preparation to form composites. Because most CDs have high stability, the subsequent steps have little impact on them, which makes this method very popular among researchers. Generally, the substrate is film [27,57], MOFs [31,32], polymer [58], or powder [34,59]. Silica is a common substrate, and some studies show that citric acid, n-(β-Aminoethyl)-γ-carbon dot nano phosphors modified by white carbon black were synthesized by one-step hydrothermal method with aminopropyl trimethoxysilane and silica as the raw materials (SiO_2_@CDs) and added it into silicone rubber to prepare silicone rubber composites with fluorescence function. Li et al. prepared CDs silica composites with room temperature phosphorescence. Using silicone resin as the matrix material, CDs implanted solid films were prepared. Teng et al. prepared CNDS and polyvinylpyrrolidone (CNDS and PVP) powder and films with different emission colors [60]. Wang thermally polymerized urea and CDs after freeze-drying, and embedded CDs into C_3_N_4_ nanotubes [61]. In the process of CO condensation, the amino group on CDs is connected with the amide bond on urea derived C_3_N_4_ to promote tubular assembly. For the preparation of organo-silane functionalized carbon dots, the prepared viscous carbon dots are dispersed in starch in the proportion of 0.5 mL/5.0 g, and the CDs/starch complex can be obtained by continuous grinding. The newly prepared viscous carbon dots are dispersed in sodium carboxymethylcellulose at a ratio of 0.5 mL/5.0 g and are continuously ground to obtain the CDs/RnOCH_3_COONa complex.

The third method is to add the prepared substrate material to the preparation process of CDs to form CDs in situ on the substrate material, so as to obtain CDs composites. This method requires that the substrate material itself has high stability or appropriate CDs preparation process. As shown in Figure 3c, the third method is to add substrate materials to CDs precursors to form a homogeneous solution, and prepare CDs composites by various methods. Substrates with high stability, such as silica or titanium dioxide materials, are more favored in this method [62]. Wang et al. loaded CDs onto amino functionalized ordered mesoporous silica (SBA-NH_2_) and prepared CDs/SBA-NH_2_ composites by the micro-plasma method [63]. SBA-NH_2_ was mixed with citric acid and ethylenediamine in water, treated by micro-plasma, and CDs was synthesized in situ. The prepared CDs/SBA-NH_2_ composite retains the fluorescence properties of CDs and the ordered pores of mesoporous silica, which may be related to the high stability of SBA-NH_2_ and moderate reaction conditions in micro-plasma.

Chung et al. mixed graphene oxide, boric acid, and glucose to synthesize CDs modified graphene hydrogel composite [64]. The obtained composites have unique three-dimensional structure and rich catalytic active sites. Liu’s team added the protein to the graphene oxide solution [65]. After hydrothermal and annealing treatment, the CDs anchored reduced graphene oxide (N, S-CDs/RGO) composite was obtained. Due to the combination of the two materials, the composites show a large number of doping sites and excellent conductivity.

The fourth method uses the one-step method to prepare CDs composites, which is suitable for more specific materials or structures. Therefore, the preparation of CDs composites is a new strategy, which can avoid the defects of CDs and bring new properties for its further application in various fields. CDs raw materials and substrate materials are mixed together to form composites (Figure 3d). This method often requires special raw materials and preparation steps, which means that it is not a general method, but a facial preparation procedure, which has attracted great attention of researchers. San et al. applied brown algae to prepare graphene oxide CDs composites with bio oil as the raw material by one-step hydrothermal method [66]. The composites showed green emission under light irradiation. They assumed that CDs were prepared from low molecular weight compounds in protein sources, while graphene oxide was prepared from high molecular weight compounds in biooil, so as to form CDs composites. In order to avoid luminescence quenching caused by aggregation, Zheng et al. prepared CDs polymer composites in one step using the hydrothermal method. CDs and polymer were carried out at the same time [67]. Some CDs were attached to large polymer particles to form CDs polymer composites without luminescence quenching. The composite powder showed a good application prospect in white LED phosphors.

## 4. Application of CDs and Composites

CDs have been widely used in biochemical sensors [29], fluorescent probes [68], biological imaging [69], photocatalytic technology [70], drug carriers [71], light emitting devices [72], energy conversion/storage devices [73], etc. Compared with traditional semiconductor quantum dots, carbon dots have low toxicity, biocompatibility, low cost, and chemical inertia, but their fluorescence properties are similar [74]. These excellent characteristics of CDs promote their applicational prospect in the biomedical field, such as fluorescence sensing and imaging in vitro or in vivo [75,76,77,78]. In the past 10 years, CDs, as a new type of fluorescent nano materials, have attracted extensive attention.

### 4.1. Biotherapy

#### 4.1.1. Photodynamic Therapy and Photothermal Therapy

**Figure 4:**
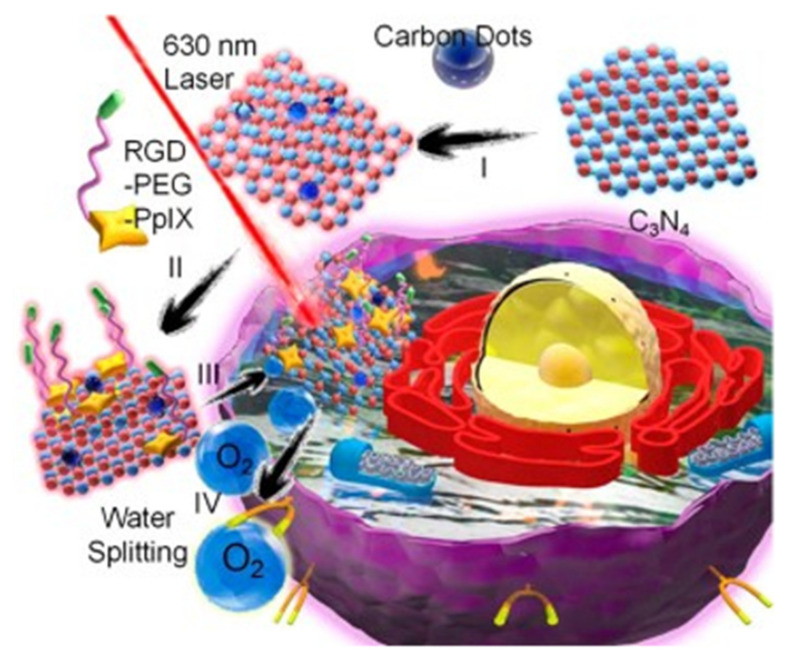
Structure of PCCN and the schematic diagram of 630 nm light-driven water splitting enhanced PDT [5] (I) carbon dot doping to prepare carbon-dot-doped C_3_N_4_; (II) PpIX-PEG-RGD self-assembly to prepare PCCN; (III) receptor-mediated endocytosis of PCCN; (IV) 630 nm light-driven water splitting to produce O_2_, in which O_2_ enhances PDT to produce ROS and kill the cancer cells.

Optical diagnosis and treatment can overcome the hypoxia of tumor tissue. Compared with traditional cancer treatment technology, it has attracted more and more attention in basic research and clinical practice in recent years, including photodynamic therapy (PDT) and photothermal therapy (PTT) because of its unique advantages of minimally invasive, high selectivity, low side effects, and low drug resistance. CDs have become a promising phototherapeutic agent because of its unique optical properties, high water solubility, and high photostability. It is reported that a hypoxic tumor microenvironment and rapid oxygen consumption during PDT will seriously hinder the therapeutic effect of CDs. In addition, the aggravation of hypoxia caused by PDT will lead to irreversible tumor metastasis or drug resistance. PDT converts oxygen into reactive oxygen species (ROS) through light irradiation and heats it with the help of a photosensitizer to induce local apoptosis of cancer cells. Zhang et al. designed CDs modified C_3_N_4_ nanocomposites for light-driven water decomposition to improve the oxygen concentration in the tumor and finally reverse the PDT resistance and tumor metastasis caused by hypoxia [79] (as shown in Figure 4). The magnetic fluorescent nano material Mn CDs prepared by Jia can also effectively generate O_2_ and further catalyze H_2_O_2_ to produce oxygen in an anoxic environment [80]. At the same time, fluorescence/magnetic resonance dual-mode imaging can improve the efficacy of PDT. PTT uses photothermal agents to generate heat by converting the energy absorbed from photons, which can locally raise the temperature and kill tumor cells. Permatasari et al. synthesized near-infrared emitting CDs with citric acid and urea, which can be used as an effective PTT therapeutic agent [5]. The photothermal conversion efficiency is more than 50%, and its unique near-infrared absorption characteristics are better than traditional photothermal agents. However, PTT usually requires high-power and long-time laser irradiation to produce enough heat to kill cells, and the highly expressed heat shock protein greatly reduces the therapeutic effect. In order to avoid the disadvantages of single mode treatment, PDT and PTT are currently used for synergistic tumor treatment [81] to jointly expand the application of CDs in the field of tumor treatment.

#### 4.1.2. Cancer Theranostics

**Figure 5:**
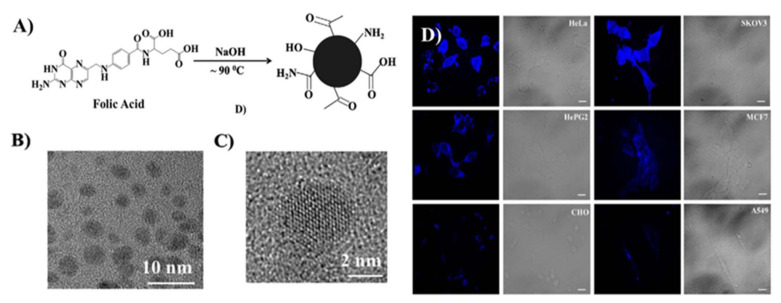
(**A**) Synthesis of the CDs from folic acid. (**B**) Transmission electron microscopy (TEM) imaging of the CDs. (**C**) High-resolution TEM (HR-TEM) image of a CDs. (**D**) CDs used for fluorescence microscopic imaging of cells expressing the folate receptor to different degrees [83], scale bars are 10 μm.

In the field of medical cancer research, it is of great significance to study the in vitro imaging of cancer cells and clarify the effect of drugs on cancer cells to study the pathological process of cancer cells. At present, we have made great efforts to determine whether cancer cells in the human body can be specifically recognized. We have mainly developed compounds with targeted recognition and selective labeling, especially fluorescent dyes, to specifically dye cancer cells, which plays an important role in the direct research on cell biology and various cell processes and therapies. In recent years, fluorescent semiconductor nanoparticles (usually called quantum dots) [82] have been widely used in cell labeling, but quantum dots are generally cytotoxic (such as element barriers and arsenic), and functional design is usually required to improve their biocompatibility; Fluorescent dyes and nanoparticles are used as markers of cancer cells. Another significant challenge is to achieve cell specificity. The method of cell targeting is usually used to design folic acid receptors that bind to cell surface receptors. Folic acid is a metabolite with the highest content in many cancer cells. Based on this, we found that fluorescent carbon dots synthesized from folic acid can be used as an effective carrier to image the surface of cancer cells expressing folic acid receptors. A foreign team used folic acid as the carbon source to synthesize carbon dots by a simple one-step method (as shown in Figure 5) [83]. The carbon dots not only have good biocompatibility, but also may be less cytotoxic than semiconductor quantum dots. The newly designed carbon dots can not only promote the recognition of folic acid receptor agonists/antagonists, but also lay a foundation for the development of new cancer treatment methods.

#### 4.1.3. Gene Therapy

At present, the most widely used clinical treatment method for cancer is chemotherapy. However, due to the large side effects of chemotherapy, researchers have explored a “diagnosis + treatment” gene therapy method based on the treatment path. This method can not only improve drug development and disease management, but also reduce the treatment risk and cost. The success of gene therapy depends on the generation of a carrier, which can effectively, selectively, and with low toxicity deliver genes to target cells and improve the health of patients with various genetic diseases, including cancer, diabetes, cardiovascular diseases, and mental disorders. The use of nucleic acids including pDNA, mRNA and non-coding RNAs as gene therapy models is rapidly entering clinical practice. Nanoparticles have received great attention as non-viral gene transfer carriers. CDs are new nano carriers for gene transfer applications due to their low toxicity, high quantum yield, low light bleaching, good water solubility, easy surface modification, and chemical stability. Liu et al. reported for the first time in 2012 [84] and Wang et al. in 2014 [85] that CDs can be used as a safe and effective imaging traceable nano carrier for in vitro and in vivo gene transfer. SG and KG reported in 2019 that carbon quantum dots (CDS) were synthesized from sweet lemon peel and coupled with different generations of polyaminoamine (PAMAM) dendrimers to obtain cd-pamam conjugates (CDPS) [86]. CDPS is a promising gene carrier system that can be used for gene therapy of triple negative breast cancer.

### 4.2. Drug Delivery

In recent years, there have been some other reviews discussing the research related to the use of CDs in cancer diagnosis and treatment [87,88], and these works play an important role in the field of cancer research, but CDs also have broader applications in tissue engineering, drug delivery and other fields. In addition to anti-cancer by optical diagnosis and treatment, CDs can also combine imaging tools with drugs to form nano hybrids for imaging guided multifunctional drug delivery, so as to improve the efficiency of drug delivery. Drug delivery is a safe and effective treatment method, which is used to carry the drug to a specific position in the body and release it continuously. Therefore, the controlled release and accurate targeting of the drug delivery system are very important to improve the local treatment effect, reduce non infectivity, and reduce side effects. Due to their fluorescence characteristics, CDs can track and observe the aggregation and activity of drugs in pathological sites in real time, which is of great significance for evaluating the efficacy of drugs [89]. By modifying a targeted biomolecule on the surface of CDs, we can actively target specific cancer cells and limit their contact with healthy tissues. Compared with passive targeting, active targeting can use the specific interaction between targeted ligands and corresponding receptors overexpressed on the surface of cancer cells to enhance the endocytosis of drug carriers, so as to achieve effective tumor therapy. Fan et al. realized real-time imaging monitoring of tumor treatment by tracking the green fluorescence generated by the combination of folic acid modified CDs (FA-CPDs) and chloroquine [90]. Similarly, Sung et al. embedded CDs and anticancer drugs in nano aggregates to achieve effective drug delivery, imaging, and photolysis for deep tumors [91]. Zhou et al. designed multiple pairs α-Red functionalized fluorescent CDs of carboxyl and amino groups that can target tumors, including gliomas, due to their multivalent interaction with neutral amino acid transporters [92], which is highly expressed in most tumors. In addition, functional CDs mimic large amino acids in structure, can load aromatic drugs through π-π stacking interaction, enable infrared fluorescence and photoacoustic imaging of tumors, and target tumors to deliver chemotherapeutic drugs. Therefore, the functionalization and high selectivity of CDs to tumors make them widely applicable to tumor specific imaging and drug delivery, and show potential clinical application prospects in the imaging and administration of central nervous system diseases.

### 4.3. Biological Imaging

**Figure 6:**
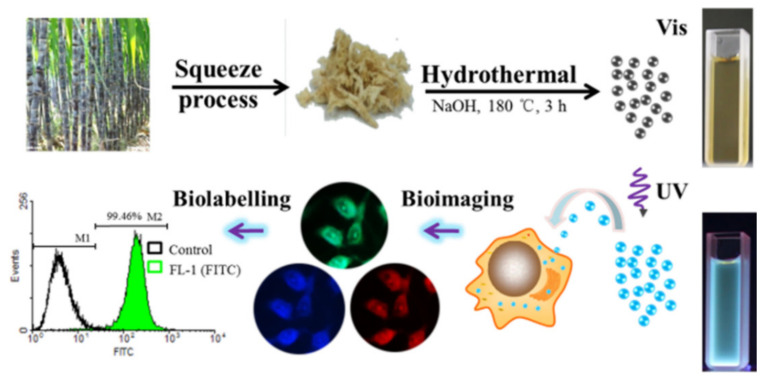
Schematic synthesis of bagasse-derived CDs from the HTC of the bagasse and the following biomedical applications [94].

Nitrogen-doped carbon dots with photoluminescence were synthesized using the hydrothermal carbonization process in an affiliated hospital, with glucose and glycine as the carbon source and nitrogen source, respectively [93]. The prepared carbon dots were mono-disperse spherical particles with a particle size of about 2.8 nm. The toxicity of A549 cells was tested by MTT method, and the cytotoxicity was very low. The doped nitrogen atoms are embedded in the crystal structure of carbon dots in pyridine configuration. It is this new internal structure that leads to the aqueous solution of nitrogen-doped carbon dots, showing wavelength-dependent polychromatic photoluminescence and good biocompatibility. As a fluorescent probe for living cell labeling, tracking, and imaging, it replaces the traditional quantum dots or molecular probes. Although the fluorescent properties of quantum dot probes are strong, the composition of quantum dots often contains toxic heavy metal ions. We used sugarcane as the raw material to prepare fluorescent carbon dots with good photoluminescence performance, high stability, and good water dispersion through heat treatment. The preparation route is shown in Figure 6, and it further proved that carbon dots derived from bagasse can be used as biomedical markers and imaging [94].

**Figure 7:**
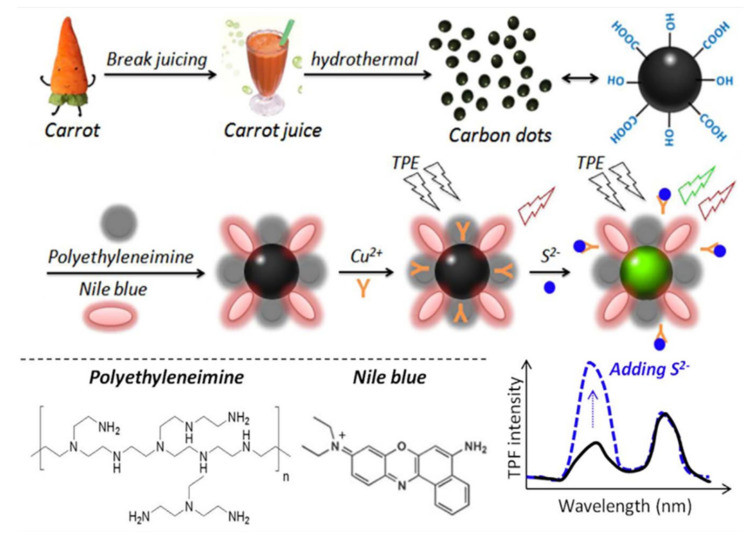
Schematic illustration of the preparation of CDs and CDs/PEI/NB nanocomposites for ratio-metric TPF turn-on sensing of S^2−^ ions [95].

CDs/PEI/NB nanocomposites were prepared in aqueous solution by the hydrothermal carbonization process using natural carrots as the carbon source and hydrothermal carbonization at 180 °C for 5 h [95]. The negatively charged -COOH and -OH remained on the surface of the carbon dots, and CDs/PEI/NB nanocomposites were prepared by combining them with positively charged polyethyleneimine (PEI) and Nile Blue (NB) in an aqueous solution, as shown in Figure 7. Under the excitation of 800 nm fluorescence, it was found that the composites had two-photon fluorescence (TPF) effect. Cao et al. observed for the first time that CDs emit visible fluorescence with TPF characteristics under 800 nm pulsed laser excitation 12. Since then, studies on TPF of CDs have also been reported [96,97,98,99]. In recent years, TPF or up-conversion fluorescence of CDs has been widely used in cell imaging [100,101,102,103].

**Figure 8:**
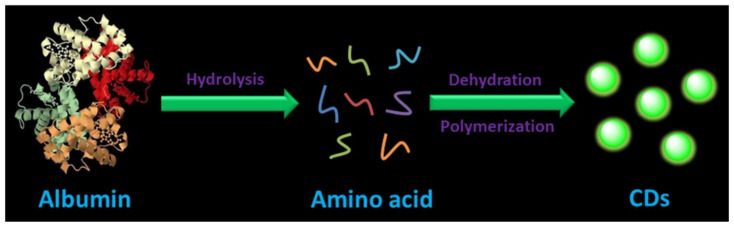
The possible mechanism of forming CDs [104].

**Figure 9:**
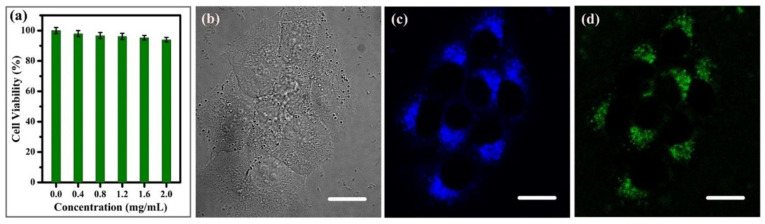
(**a**) The effect of CDs on Bcap-37 cell viability (MTT assay). Confocal fluorescence microphotographs of CDs labeled Bcap-37 cells under a bright field (**b**) at the excitation wavelengths of 405 nm (**c**) and 488 nm (**d**). All scale bars are 20 μm [104].

Hu et al. prepared high fluorescence carbon dots at room temperature using the natural green synthesis method [104]. CDs were synthesized by microwave (MW) and ultrasonic operation (UM) with albumin (egg white) as the carbon source and different amounts of NaOH. The possible forming mechanism of CDs is shown in Figure 8. Under strong alkali conditions, albumin first forms a single amino acid with an extremely high concentration through complete hydrolysis [105], and then amino acids as the precursor materials get organic aggregates through dehydration condensation, and finally form CDs through intramolecular and intermolecular dehydration. MTT assay was carried out to evaluate the cytotoxicity of CDs, as shown in Figure 9. First, Bcap-37 cells were incubated in different concentrations of carbon dots for 24 h to detect their viability (Figure 9a). The extract was dissolved in the culture dish inoculated with breast cancer Bcap-37 cells, and the cells were imaged under the excitation of a laser with wavelengths of 405 and 488 nm emitting blue and green fluorescence, respectively (Figure 9c,d), and a cytotoxicity test was carried out. The fluorescence dependent emission behavior was confirmed by biological cell imaging. The results showed that CDs had excellent biocompatibility, and it was explored that the emission of CDs originated from the emission of defective states and eigenstates. It can be used for in vivo cell imaging and biological probes.

### 4.4. Biosensor

**Figure 10:**
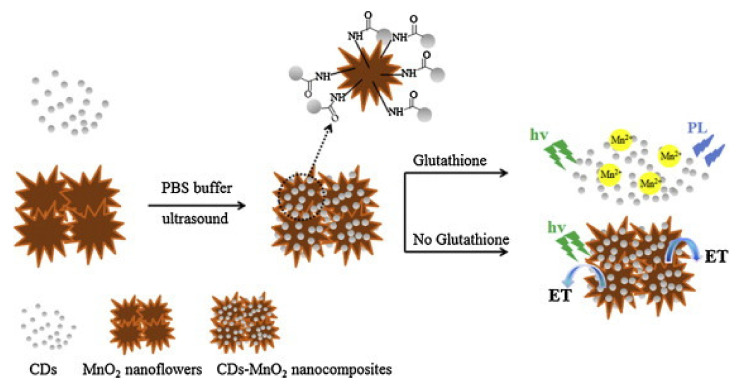
Schematic diagram of GSH detection of CD-MnO_2_ nanocomposites [8].

Human health is inseparable from the role of biomolecules in the body. In recent years, for the monitoring of biomolecules in vivo, a simple and reliable CDs composite sensor for biosensor has been developed [106,107]. Studies have shown that glutathione (GSH) and cysteine (Cys) are molecules that can prevent reactive oxygen species from damaging cellular components. Wu et al. used the “on-off” strategy to establish a fluorescent nano probe of N-doped CDs for the sensitive detection of GSH [108,109]. The luminescence of CDs has an inverse relationship with GSH concentration because sulfhydryl triggers the inner filtering effect (IFE). Cai [110,111] and others designed a new “on-off-on” biosensor using CD-MnO_2_ nanocomposites for GSH sensing. In this system, the photoluminescence (PL) of CDs was first quenched by MnO_2_ nanosheets through the energy transfer; in the presence of the target analyte GSH, the manganese dioxide nanosheets were reduced to Mn^2+^ ions, resulting in the release of CDs and the recovery of CDs PL (Figure 10) [8].

**Figure 11:**
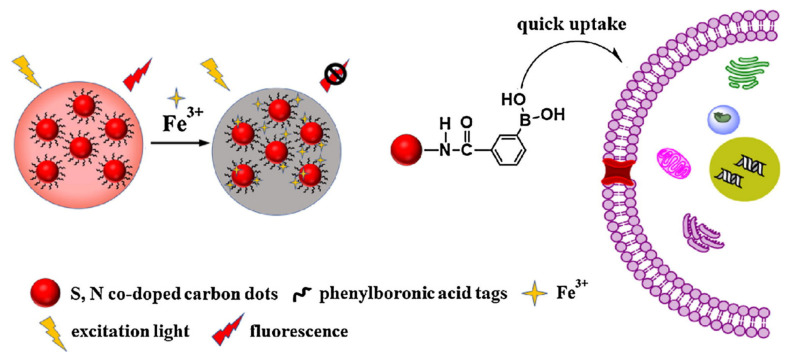
Schematic diagram of N, S-CDs-PBA sensing Fe^3+^ in PC12 cells [8].

As serum alkaline phosphatase (ALP) is an important biological molecule, which is closely related to cancer, bone disease, and other diseases, the determination of ALP is also very important [112]. Li et al. established a sensing platform for N-doped CDs to detect ALP quickly and sensitively [113]. In the composite, CDs and p-nitrophenyl phosphate (PNPP) were converted to p-nitrophenol (PNP) under ALP catalysis. Since the emission of CDs and the absorption of PNP overlap well, PNP can effectively quench the PL of CDs through IFE, so as to realize the sensing of ALP. Nucleic acids are also biologically important molecules. Therefore, the nucleic acid detection and sensing platform designed based on the CD composite has also been widely studied [114,115]. For example, Somaye et al. developed an ultra-sensitive homogeneous biosensor for detecting HIV-related DNA sequences, which was realized by FRET between CDs and AuNPs [116,117]. Some other studies [118] used Cd Te-CDs as the probe, mitoxantrone as “on-off-on” signal reagent, and also used the PET mechanism to detect DNA. In addition to the above [119,120,121,122,123,124,125,126,127], the CD composite was also used as a probe to successfully realize the sensing of ATP, trypsin, dopamine, glucose, RNA, and other biologically important molecules. Li et al. reported a CDs (N, S-CD) sensor based on N and S co-doping, which is used to sensitively detect mercury in living cells. Its sensing is realized by PL quenching of N and S-CDs caused by electron transfer and coordination interaction between N and S-CDs [128]. According to the same method, Zhang et al. successfully realized the sensing of Fe^3+^ ions, in which N and S co-doped CDs were labeled with phenylboric acid (N, S-CDs-PBA) [129]. Fe^3+^ can coordinate with phenolic hydroxyl and carboxyl groups on the surface of CDs, so as to transfer the excited electrons of N, S-CDs-PBA to Fe^3+^, causing fluorescence quenching (Figure 11).

### 4.5. Capacitor

**Figure 12:**
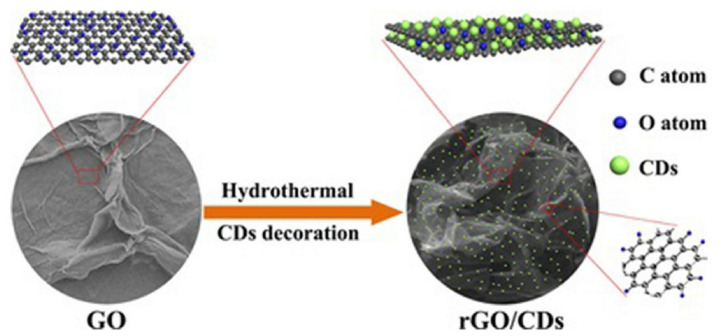
Schematic diagram of the synthesis process of three-dimensional interconnected CD decorative reduced graphene oxide nanosheets (rGO/CDs) [9].

Among the many energy storage devices, supercapacitors have the characteristics of high energy density, fast charge and discharge, durability, and so on [130,131,132]. CDs composite can be used as an important part of a supercapacitor electrode. Carbonaceous porous materials and porous metal compounds have a large specific surface area, which can store more carriers and improve the capacitance. The abundant functional groups on the surface of CDs can provide more active sites. For example, the composite material prepared by introducing colloidal quantum dot (CQD) into carbon aerogel is an excellent electrode material for supercapacitors [133]. The introduction of CQDs provides additional active sites, while the retained interconnected nanochannels promote ion diffusion and shorten the charge transport path. Li et al. proposed N, P-CQDs/reduced graphene oxide (rGO) composite aerogel as the supercapacitor electrode material [134]. In addition to the contribution of layered porous structure and CDs to the excellent electrochemical performance of supercapacitors, N and P co-doping can also provide more active sites, increase the specific surface area and enhance the electronic conductivity, so as to improve the capacitance. The introduction of CQDs helps improve the conductivity of CQDs/Ni(OH)_2_ composites, promote the formation of the mesoporous structure, and improve their electrochemical properties. Zhao et al. reported that the super capacitor based on 3D interconnected CDs modified rGO nano sheet (rGO/CDs) has excellent electrochemical performance [9] (Figure 12).

### 4.6. Electrocatalysis

**Figure 13:**
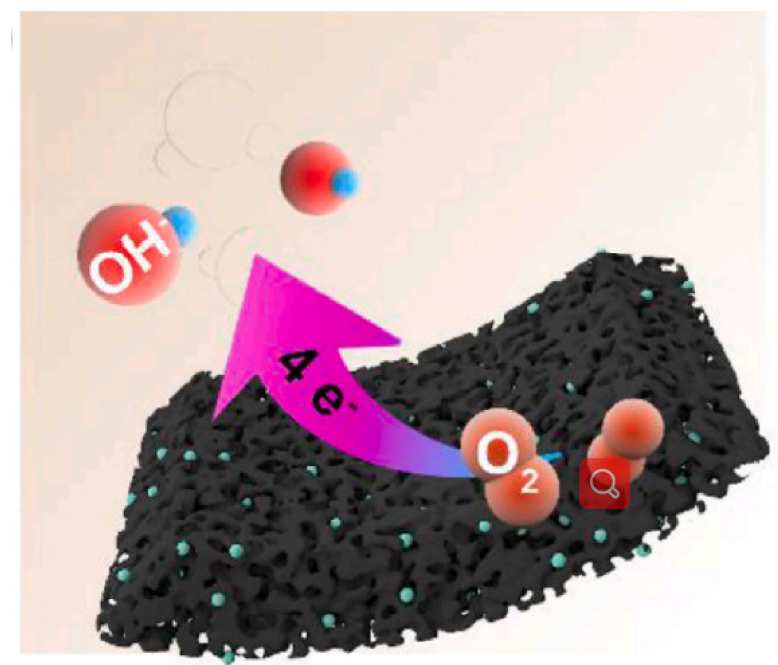
Mechanisms of CDs/NSPC for ORR [137].

**Figure 14:**
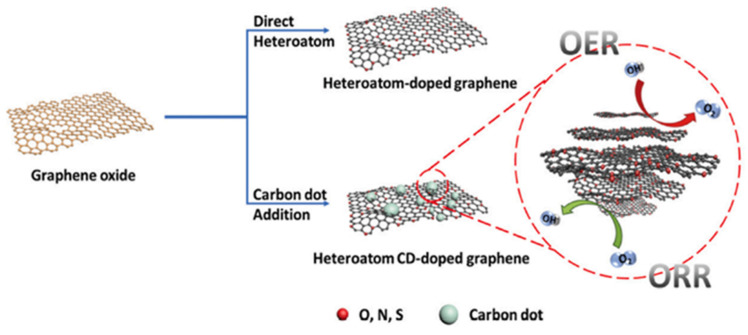
The schematic preparation process of NS-CD@gf [10].

CDs have gradually become a potential substitute for traditional noble metal catalysts because of its good conductivity and high stability, as well as the ability to improve the electrocatalytic performance by doping nitrogen, phosphorus, and other atoms. Adding CDs into porous materials can effectively prevent the aggregation of CDs and provide more active sites, so as to show better electrocatalytic performance. For example, Zhang et al. prepared N-CNDs@MCNSs composites by coating CNDs on acid-treated for oxygen reduction reaction (ORR) [135]. Due to its high water dispersion, a single N-CNDs cannot form a stable ORR catalytic electrocatalyst membrane. N-CNDs@MCNSs, as an ORR electrocatalyst coated on the bare GC electrode, has high electrocatalytic activity, which is very close to the starting potential of Pt/C catalyst and shows a four electron ORR process. Under the same stability test conditions, the retention rate of the catalytic activity of coated electrode (more than 90%) was higher than that of the Pt/C coated GC electrode (76%). NDs/MCNSs have high ORR catalytic activity and good operational stability, because MCNSs can stabilize and disperse N-CNDs to improve electrocatalytic performance. CDs self repair nitrogen sulfur co-doped porous carbon composite (CDs/NSPC) is another electrocatalyst designed by Liang et al. [136]. For ORR catalysis. The rich edge of CDs endows the catalytic activity of ORR. Heteroatom doping can further improve the catalytic activity. The porous structure of CDs/NSPs can provide more active sites. As a result, the electrocatalyst showed significant ORR catalytic performance in alkaline medium, which was better than Pt/C, NSPC, and NSC, as shown in Figure 13 [137]. Recently, Shin et al. prepared carbon-based bifunctional electrocatalysts (NS) of ORR and OER using the hydrothermal method and annealing treatment (NS-CD@gf). Optimal NS-CD@gf has ORR activity superior to conventional Pt/C and OER activity equivalent to Ir/C (Figure 14) [10]. The study found that NS-CD@gf The ORR and OER activities are derived from the heteroatom functional groups of CDs. The porous carbon framework can promote the migration of oxygen molecules, and the two cooperate to improve the electrocatalytic performance.

### 4.7. Photocatalysis

**Figure 15:**
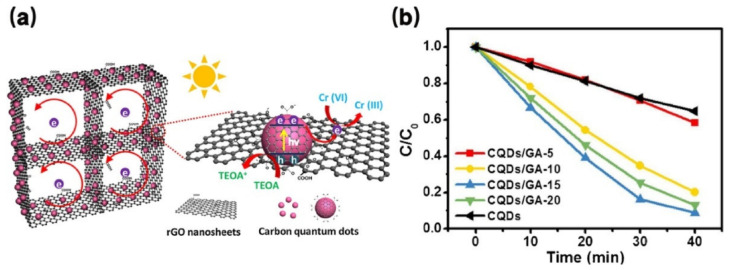
(**a**) Photocatalytic mechanism of the 3D CQDs/GA composites. (**b**) Photocatalytic reduction of Cr (VI) with different proportions of CQDs/GA and original CQDs [4].

As an environmentally friendly and effective method of producing energy and decomposing pollutants, photocatalytic technology has recently attracted more and more attention [138]. CDs have the ability of photo excitation to produce hole electron pairs and have potential application prospects in the field of photocatalysis. However, the photocatalytic activity of single component CDs is low because the photogenerated carrier cannot be effectively separated. The assembly of CDs with high conductivity porous materials such as carbonaceous porous materials can significantly improve the photocatalytic performance. For example, Wang et al. developed a metal-free catalyst by dispersing CQDs in graphene aerogel [4]. CQDs can be used as a photosensitizer to generate photogenerated carriers. The unique porous structure of graphene aerogel promotes the separation of photogenerated carriers and exposes more active sites (Figure 15a) [4]. Through synergy, CQDs/citric acid (GA) composites have enhanced photocatalytic activity. After 40 min of UV-Vis irradiation, the optimal CQDs/GA-15 removed 91.3% of Cr (VI), about 2.6 times that of the original CQDs, as shown in Figure 15b [4].

**Figure 16:**
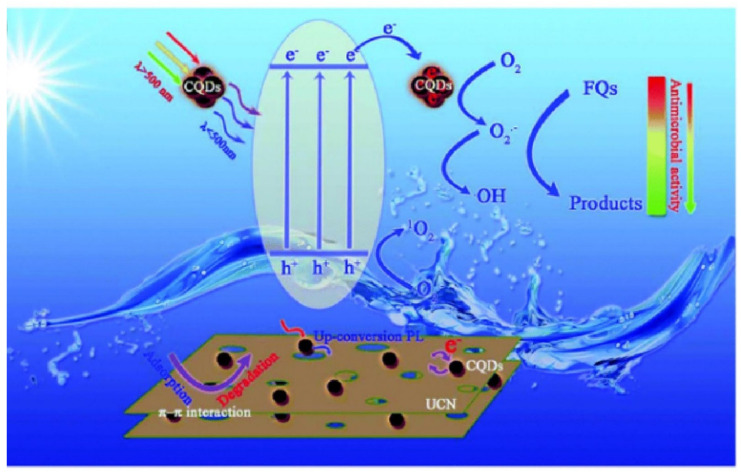
Mechanism of photocatalytic degradation of OFX by mpg-C_3_N_4_/CQDs [139].

In addition, CDs can also enhance the photocatalytic performance of semiconductor catalysts through their electron transfer, up conversion, and down conversion luminescence characteristics. For example, Wang et al. coupled CQDs with mesoporous g-C_3_N_4_ to prepare high-efficiency photocatalysts (mpg-C_3_N_4_/CQDs) [139]. Firstly, the adsorption performance of the photocatalyst was investigated. Due to its porous structure, the mpg-C_3_N_4_-related photocatalyst has stronger adsorption capacity for different substrates than g-C_3_N_4_, which is helpful to improve the photocatalytic performance. In addition, the photocatalytic activity of mpg-C_3_N_4_/CQDs was tested by degrading ofloxacin (OFX) under visible light irradiation. At the same time, the degradation rate of OFX by mpg-C_3_N_4_/CQDs was 90.1%, which was significantly higher than that of g-C_3_N_4_ (8.3%) and mpg-C_3_N_4_ (65.6%), indicating that CQDs played an important role in the degradation of OFX. The mechanism of photocatalytic degradation of FQs by mpg-C_3_N_4_/CQDs is proposed, as shown in Figure 16. Under visible light irradiation, light with a wavelength below 500 nm can be directly absorbed by mpg-C_3_N_4_, while light with a wavelength above 500 nm can be converted into light below 500 nm by CQDs and further absorbed by mpg-C_3_N_4_, resulting in more electron hole pairs. The photogenerated electrons on mpg-C_3_N_4_ are transferred to CQDs, resulting in the effective separation of photogenerated carriers. Finally, O_2_ is generated by photogenerated electron reaction and used to decompose OFX adsorbed by mpg-C_3_N_4_/CQDs. Similarly, Shen et al. [140] prepared CQD/p-InVO4 nanocomposites, which degraded Rhodamine B (RhB) by simple reflux method and had enhanced photocatalytic performance. During the degradation of RhB, the optimal photocatalytic decomposition rate of CQD/p-InVO4 nanocomposites was 0.085 H^−1^, which was 2 and 1.25 times higher than that of p-invo4 and commercial catalysts, respectively. CQD improves the absorption of p-InVO4 to sunlight and effectively transfers photogenerated electrons, while the porous structure of CQD/p-InVO4 nanocomposites can adsorb more RhB and improve its photocatalytic degradation ability.

### 4.8. Genotoxicity

In general, the toxicity of CDs in the application of biomedical field is very low. When studying the biological effects of nanomaterials, genotoxicity is also an important parameter to be tested. Although carbon nanomaterials may not affect the genome, they may be harmful to other parts of the cell. In 2019, Senel et al. synthesized a new type of nitrogen-doped graphene quantum dots with citric acid and p-aminophenol as raw materials using the hydrothermal method, and measured the genotoxicity using the comet method. The results showed that nitrogen-doped graphene quantum dots could be internalized into cells quickly, and undamaged DNA would not lose its integrity [141]; In 2022, Saikia et al. prepared two fluorescent emission nitrogen and sulfur co-doped carbon quantum dots (NS CQDs) with sub bituminous coal as the precursor using the ultrasonic wet chemical process. They evaluated the genotoxicity of coal source CQDs using rodent liver cell lines. When cells were treated with a certain concentration of coal source CQDs for 24 h, compared with standard cells, there was no DNA damage in the treated cells, indicating that the carbon dots did not have genotoxicity [142].

## 5. Conclusions

The current review provides a broad understanding of carbon dots, carbon dot composites, and the application of carbon dot composites. CDs are a new kind of carbon-based nano material with a diameter of less than 10 nm. This review consists of three parts. In the first part, the “top-down” method and “bottom-up” method and corresponding synthesis methods of carbon dots are mainly summarized. The “top-down” is to form smaller nanoparticles by stripping or etching larger carbon materials (graphite, graphene, carbon nanotubes, carbon black, coal, etc.) The synthesis strategy is simple and efficient, but in the specific synthesis process, it is difficult to accurately control the experimental conditions, resulting in a wide range of carbon dots. The “bottom-up” synthesis strategy usually produces carbon dots with amorphous carbon nuclei, rich doping sites, and many surface functional groups. The second part mainly introduces the preparation process and methods of carbon point composites, which can be briefly summarized as “one-step synthesis” and “two-step synthesis”. The preparation process of CDs by “one-step synthesis” is simple and more suitable for the preparation of composites with specific properties and structures. CDs composites prepared by “two-step synthesis” can protect the stability and initial properties of CDs and substrate materials, so they have been widely used. In the third part, the applications of carbon dot composites in biomedicine, biosensor, electrochemistry and photocatalysis are summarized. In addition, CDs composites still have great potential for exploration and broad application prospects in the biomedical field, which is worthy of further research, in order to develop more novel functions and applications in a wider range of fields in the future.

CDs may provide better strategies in the design of biomedical devices and implant coatings due to their unique nano properties and fluorescence properties. At present, nanomaterials have played an increasingly important role in drug-controlled release, targeted drug delivery, and tissue repair after device implantation. However, after implantation, the characterization of some important subsequent phenomena is still very limited, such as the distribution of delivered drugs in tissues and cells, the metabolic pathway, the matching degree of drug release and tissue repair, and the interactions of released drugs with different cells. The research on CDs is expected to provide new perspectives and research methods in these areas.

## Data Availability

Not applicable.
